# Nurse managers' perceptions and experiences regarding staff nurse empowerment: a qualitative study

**DOI:** 10.3389/fpsyg.2015.01585

**Published:** 2015-10-14

**Authors:** Peter Van Bogaert, Lieve Peremans, Marlinde de Wit, Danny Van heusden, Erik Franck, Olaf Timmermans, Donna S. Havens

**Affiliations:** ^1^Division of Nursing and Midwifery Sciences, Faculty of Medicine and Health Sciences, Centre for Research and Innovation in Care, University of AntwerpAntwerp, Belgium; ^2^Department of Nursing, Antwerp University HospitalAntwerp, Belgium; ^3^Department of Health Care, Karel de Grote University College AntwerpAntwerp, Belgium; ^4^Academia for Welfare and Health, HZ University of Applied Sciences VlissingenVlissingen, Netherlands; ^5^School of Nursing, The University of North Carolina at Chapel HillChapel Hill, NC, USA

**Keywords:** nurse managers, structural empowerment, quality of care, patient safety, qualitative study

## Abstract

**Aim:** To study nurse managers' perceptions and experiences of staff nurse structural empowerment and its impact on the nurse manager leadership role and style.

**Background:** Nurse managers' leadership roles may be viewed as challenging given the complex needs of patients and staff nurses' involvement in both clinical and organizational decision-making processes in interdisciplinary care settings.

**Design:** Qualitative phenomenological study.

**Methods:** Individual semi-structured interviews were conducted with 8 medical or surgical nurse managers in a 600-bed Belgian university hospital between December 2013 and June 2014. This hospital was undergoing conversion from a classical hierarchical, departmental structure to a flat, interdisciplinary model.

**Results:** Nurse managers were found to be familiar with the structural empowerment of clinical nurses in the hospital and to hold positive attitudes toward it. They confirmed the positive impact of empowerment on their staff nurses, as evidenced by increased responsibility, autonomy, critical reflection and enhanced communication skills that in turn improved the quality and safety of patient care. Structural empowerment was being supported by several change initiatives at both the unit and hospital levels. Nurse managers' experiences with these initiatives were mixed, however, because of the changing demands with regard to their manager role and leadership style. In addition, pressure was being experienced by both staff nurses and nurse managers as a result of direct patient care priorities, tightly scheduled projects and miscommunication.

**Conclusion:** Nurse managers reported that structural empowerment was having a favorable impact on staff nurses' professional attitudes and the safety and quality of care in their units. However, they also reported that the empowerment process had led to changes in the managers' roles as well as daily practice dilemmas related to the leadership styles needed. Clear organizational goals and dedicated support for both clinical nurses and nursing unit managers are imperative to maintaining an empowering practice environment which can ensure the best care and healthy, engaged staff.

## Introduction

A growing body of evidence suggests that a balanced, healthy, and supportive nurse practice environment is a crucial condition for achieving and maintaining a stable, engaged and productive nurse workforce (Rafferty et al., 2001; Estabrooks et al., 2002; Choi et al., 2004; Vahey et al., 2004; Gunnarsdóttir et al., 2007; Li et al., 2007; Schubert et al., 2009; Havens et al., 2013). Such an environment is also associated with favorable patient outcomes such as relatively low mortality and co-morbidity rates and fewer adverse events (Tourangeau et al., 2005; Laschinger and Leiter, 2006; Aiken et al., 2008; Friese et al., 2008). Various studies have identified the effect that empowerment in nurse practice environments can have on nurse and patient outcomes, based on ideas described by Kanter (1993), which define empowerment as nurses' ability to mobilize all necessary resources, both human and material, to support the best care for their patients. In an empowered practice environment, nurses have access to the information they need and opportunities for learning, which stimulates personal development and fosters supportive relationships with supervisors, peers and subordinates. Informal and formal networks of alliances within the organization provide nurses with opportunities to achieve their goals and ensure professional discretion and visibility. Kanter thus described empowerment structurally, whereas Spreitzer (1995) considered it a psychological response to conditions within the practice environment which lead nurses to experience a certain degree of *meaning, competence, self-determination, and impact*.

Various studies have described the effect that conditions for nurse structural empowerment have on the experience of empowerment (Laschinger et al., 2004, 2014; Wagner et al., 2010; Yang et al., 2013; Wang and Liu, 2015). Research links nurse structural and/or psychological empowerment with job satisfaction, commitment, engagement, and spirit at work, as well as work effectiveness, unit effectiveness and quality of work (Laschinger et al., 2004, 2009, 2014; Wagner et al., 2013; Yang et al., 2013; Eo et al., 2014; Wang and Liu, 2015). Most prior studies have also revealed positive associations between staff nurses' health and wellbeing and patient care quality, safety, and mortality rates.

Tourangeau et al. (2007) reported counterintuitive findings, however: nurse practice environment characteristics, staffing adequacy and staff qualifications were indeed found to be related to lower mortality rates, but also to higher staff nurse burnout rates. The authors argued that staff nurses who became aware of potentially critical patients as a result of timely and accurate observations and treatment might also experience more work-related stress. Similar results were seen in reports and studies associated with one of the National Health Service (NHS) Institute of Innovation and Improvement's largest quality improvement initiatives, namely the Productive Ward–Releasing Time to Care™, following 10 years of development and implementation: a review reveals positive experiences and outcomes for patients and staff nurses but also negative nurse outcomes (White, 2015). The author reports that nurses and ward-based teams found it difficult to find time in busy clinical environments for improvement activities with no additional resources. Moreover, the review reported that, because of such difficulties, some Productive Ward sites were temporarily “suspending” their quality improvement activities in order to deal with the various crises that routinely affect wards and organizations.

Our previous research results have demonstrated that characteristics of nurse empowerment, such as balanced workload, decision latitude and social capital, are associated with job satisfaction, intention to leave the hospital and the nursing profession and favorable nurse-assessed quality of care (Van Bogaert et al., 2013). These empowerment characteristics appear to play a meditating role between the nurse practice environment described by the nurse-physician relationship, nurse management at unit level and hospital management on the one hand and outcome variables on the other. Further, analyses indicated associations among study variables at nursing unit level (Van Bogaert et al., 2014a) which highlighted shared experiences within units and the importance of nursing teams. Davies et al. (2011) showed the pivotal role played by nurse managers in supporting empowering work environments that are conducive to knowledge transfer in practice and evidence-based care. While Skytt et al. (2014) found that all managers described staff access to empowering structures as important, not all managers reflected on and discussed empowerment as a strategic issue.

A longitudinal study, conducted in a 600-bed university hospital, investigated the effect of the hospital's transition from a hierarchical, departmental structure (see Method section; Study context) to a flat, interdisciplinary model across three measurement periods (2006, 2011, 2013) (Van Bogaert et al., 2014b). During the study period, nurses assigned to 21 clinical units were found to perceive improved nurse practice environments as being associated with improved outcome variables. These results demonstrated the importance of a nurse practice environment characterized by good interdisciplinary relationships and support from peers, supervisors, nurse administrators, and executives within a structure of shared values and shared governance (e.g., structural empowerment) in which nurses' daily practice is focused on quality patient care.

A qualitative study was subsequently conducted in order to gain a better understanding of the organizational evolution taking place in the study hospital and of the role played by structural empowerment in achieving better patient and staff nurse outcomes (e.g., health and professional well-being).

## Methods

### Aim

To study nurse unit managers' perceptions and experiences of staff nurse structural empowerment and its impact on managers' leadership roles and styles.

### Design

We used a phenomenological method within a constructivist paradigm, starting from the nurse managers' perspective, in order to reveal essential general meaning structures surrounding particular phenomena. One investigator performed individual semi-structured interviews, a technique appropriate to the phenomenological approach.

### Study context

In 2007, the study hospital's management decided to transform the way in which the hospital was organized to more accurately address complex patient needs in the continuously evolving healthcare and socioeconomic contexts (Van Bogaert et al., 2014b). The aim of the transformation process was to develop a flatter, more interdisciplinary structure to replace the existing hierarchical, departmental structure. The overall goal was to improve the involvement and empowerment of nurses and other healthcare workers in order to cope with changing patient needs and thus provide the best care. Inspired by the American Nurses Credentialing Centre's (ANCC) Magnet Hospital Recognition Program™ [(American Nurses Credentialing Center (ANCC), 2005, 2014; Wolf et al., 2008)] and previous studies (Wolf et al., 1994a,b; Havens and Aiken, 1999; Wolf and Greenhouse, 2006), the hospital chose 3 of the original 14 *forces of magnetism* to guide the transformation: (1) flat organizational structures in which unit-based decision-making prevails, with sufficient nurse representation in the organizational committee structure; (2) a participative management style incorporating sufficient feedback from staff nurses and the presence of visible and accessible nursing leaders; and (3) positive interdisciplinary relations with mutual respect amongst all disciplines. Several structural empowerment initiatives were implemented on the basis of these three principles (see Table 1a).

**Table 1a:** Hospital transformation process^*^.

**Initiatives**	**Outset**	**Content**	**Implementation strategy**
**TRANSFORMATIONAL LEADERSHIP**			
Strategic nursing plan	2007	Nursing mission, vision, values, and strategic plan	Development based on meetings at leadership level and unit level between CNO, nurse leaders, nurse managers, staff nurses and midwives
Leadership program	2008	Introduction and continuation program with various topics such as how to coach a team and individual team members; project management; and organizational, quality and safety strategies and policies	Training and coaching sessions for unit nurse managers and nurse leaders
**STRUCTURAL EMPOWERMENT**			
Structural support for clinical units	2009	Quarterly unit meetings; agenda set through team member consultation; setting of annual unit goals; meetings between staff nurses and physicians about specific unit-level topics	Policy development; implementation process based on meetings with CNO and nurse managers
Nursing councils	2008	Programs for transitioning newly introduced staff nurses developed by nurse preceptors	Setting goals, strategies, and implementation of programs; training programs for nurse preceptors
	2010	Programs to enhance patient safety and infection control in nursing practice and clinical units developed by dedicated champions	Setting goals, strategies, and implementation of programs; training programs for champions

* *Most relevant initiatives, not exhaustive*.

In 2012, *the Productive Ward—Releasing Time to Care*™ program was introduced in the study hospital. The aim of this program was to support improvements in clinical unit care delivery as part of the hospital transformation process. Productive Ward—Releasing Time to Care™, also known as *the productive ward*, was developed by the NHS Institute for Innovation and Improvement and launched in England in 2007. The program modules support the review of processes for identifying and eliminating activities which have no added value for patients (i.e., eliminating waste). The program's aim is to improve patient satisfaction through increased provision of care by staff and thus improve clinical and safety outcomes. The *productive ward program* supports structural empowerment through three foundational modules (see Table 1b). At the same time, many other change initiatives were being implemented in the hospital during the study period, as was an accreditation process (Joint Commission International [JCI]), as part of hospital accountability to various stakeholders such as the government and the public (e.g., patients).

**Table 1b:** **Productive ward—releasing time to care™**.

**Initiatives**	**Outset**	**Content**	**Implementation strategy**
**TRANSFORMATIONAL LEADERSHIP**			
General introduction	2012	Introduction of the productive ward program	Information sessions at hospital level for staff nurses, physicians and other healthcare workers
Lean leadership	2012	Introduction of lean philosophy, productive ward program, and leadership roles	Training and coaching sessions for nurse managers and nurse leaders
Visit pyramid	2012	Unit walk around by CEO, CNO, HR manager, and nurse leaders	Monthly (CNO, nurse leaders) and quarterly (others) scheduled
**STRUCTURAL EMPOWERMENT**			
Foundation module—*knowing what and how we are doing*	2012	Unit vision—setting, measuring and evaluating unit's key performance indicators	
Foundation module—*the well-organized ward*	2012	Unit level assessment of nurse practice environment and working conditions	Unit-level training and coaching sessions; unit meetings and workshops
Foundation module—*patient status at a glance*	2013	Unit-level patient status- overview of key characteristics	

### Study participants

All nurse unit managers on medical and surgical units (*n* = 15) at the study hospital were invited to participate in the study by the study investigator. This purposive sample of nurse managers from medical and surgical units enabled us to explore manager perceptions and experiences within relatively similar nurse practice environments. Nurse managers from intensive care units, operating theaters, emergency care units, pediatric care units and maternity units were therefore excluded from the sample. Data were collected until saturation was obtained on each research topic (*n* = 8). The investigator conducted the semi-structured interviews between January and March 2014.

### Procedures and data analyses

The semi-structured interviews were organized around four topics: the definition of staff nurse empowerment; how structural empowerment was being implemented in the unit; how it had impacted internal hospital governance and policy; and its impact on nurse unit managers' roles (see Table 2). A standardized procedure (e.g., introduction, interview, ending) guided each interview and participants also completed a short questionnaire about their demographic characteristics. All interviews were audio recorded and the investigator also took notes during the interviews.

**Table 2:** **Semi-structured interview: topics and items**.

**Topic**	**Items**
Definition	What do you understand by empowerment?
	What do you think about the principles of empowerment?
Hospital implementation	How is empowerment reflected in internal hospital governance and policy?
	What is your opinion of empowerment as a part of hospital policies?
	To what extent does empowerment support the hospital decision-making processes?
Internal hospital governance and policy	How is empowerment represented on your unit?
	What are staff nurses' opinions of empowerment?
	To what extent are staff nurses involved, satisfied and resilient?
The nurse manager's role	How does empowerment impact your role as a nurse manager?
	Did you have prior experience with staff nurse empowerment?
	To what extent did you have to change your role as a nurse manager to be aligned with the hospital policy on empowerment?
	To what extent do you think your role as nurse manager is aligned with the hospital policy on empowerment?
	To what extent are you involved and satisfied?
	To what extent does empowerment support quality of care and patient safety?
	What is your experience with empowerment on your unit?

The study investigator and a second investigator each coded a written interview independently and then developed a codebook together. Using this codebook, the study investigator coded seven interviews directly from interview recordings.

The analysis was completed using the descriptive phenomenological approach. First, we looked at the particular experiences of each of the participants and then we explored the themes common to their experiences. Various themes were identified, linked, and described. This qualitative thematic data analysis was supported by NVivo 10 software (QRS International).

### Rigor

Credibility and confirmability were achieved by the coding procedure outlined above, in which two researchers analyzed a written interview independently and subsequently developed a codebook together. The use of verbatim quotations also ensured that the participants' voices can be heard in the study (Polit and Beck, 2010; Halcomb et al., 2013).

## Results

The demographic characteristics of the participants are summarized in Table 3. Four themes were identified on the basis of thematic analyses of eight study participants' interviews, originally guided by the four topics mentioned above: *vision of empowerment*; *structural empowerment policy*; *the nurse manager's role*; and *suggestions by nurse managers to improve the empowerment policy*.

**Table 3:** **Study population demographic characteristics**.

	**Mean (range)**
Age	44 (28–55)
Years in current hospital	23 (7–34)
Years as nurse manager	14 (2–29)
	**N**
**NURSING UNIT**	
Medical unit	3
Surgical unit	4
Mixed unit	1
**QUALIFICATIONS**	
Bachelor degree in nursing	8
Master degree in nursing	3
**ADDITIONAL TRAINING**	
Management and leadership	5
Other	1

*N = 8*.

### Vision of empowerment

With the exception of two, all study participants clearly understood and were able to explain their vision of structural empowerment. Structural empowerment was described as joint decision-making among staff nurses and other healthcare workers from all organizational levels as well as involvement in internal hospital governance and policy. Empowerment was felt to influence staff nurses' shared vision to achieve their goals more autonomously and also to support quality improvements, knowledge transfer processes and evidence-based practices.

“*Empowerment to me is first and foremost the involvement of co-workers, …more power, more ability to make decisions…involvement at all organizational levels” (Interviewee 3)*.

“*Empowerment means involvement in internal hospital governance and policy and daily practice” (Interviewee 5)*.

“*To move people toward a shared vision…I think all efforts to improve quality of care on your unit” (Interviewee 6)*.

“*To accumulate knowledge about our hospital and ourselves, to align our jobs with current norms and evidence-based practices” (Interviewee 8)*.

### Structural empowerment policy

Nurse managers expressed both positive and negative perceptions and experiences of structural empowerment. They felt that certain change initiatives and projects did support staff nurse empowerment and involvement in decision-making processes, such as nurse champions, the productive ward program, nursing councils, nurse preceptors and accreditation/credentialing organized by JCI and the ANCC Magnet Recognition Program. Nurse champions and the productive ward were primarily seen as unit-level initiatives. Nurse preceptors had already existed for many years, but some changes had been noticed.

“*For example nurse preceptors have certain responsibilities to provide nursing unit colleagues with information and to encourage them to participate and collaborate” (Interviewee 3)*.

“*Because of various projects such as the productive ward …major contributions were expected of nurses” (Interviewee 5)*.

“*The nurse preceptor role has existed for many years but it has recently become more embedded” (Interviewee 2)*.

“*There is awareness about JCI accreditation, because of ward rounds, but it is still experienced by staff nurses as remote. The idea of Magnet Hospital recognition is more visible at leadership level than at staff nurse level” (Interviewee 4)*.

“*We also have nursing councils with staff nurse membership—our champions—through which nurses' voices have been heard” (Interviewee 1)*.

The study participants unanimously described structural empowerment as positive. Moreover, internal hospital governance and structural empowerment were viewed as good and necessary. Staff nurse involvement and joint decision-making were being stimulated through various projects, and staff nurses were becoming more responsible.

“*They listen more …to staff nurses' concerns and issues …a certain strategy in which staff nurses were involved” (Interviewee 3)*.

“*Everyone has the feeling that they can contribute. It doesn't always come from the top down” (Interviewee 5)*.

Because of these change initiatives, nurses' work has had to become more structured, uniform and transparent across procedures and communication strategies. The changes also supported improvements in patient care quality and efficiency. Staff nurses were engaged and able to reflect critically on the quality of their care.

“*We have to do things by ourselves. Information is provided but we have to search for the right information by ourselves. That is our new culture …” (Interviewee 5)*.

Nurse managers recognized that patient care and safety improvements had come about through aspects of structural empowerment and various change initiatives and projects (e.g., critical reflection, standardization of work, etc.). Some nurse managers did report negative perceptions and experiences of staff nurse empowerment, however.

“*Too many expectations and too much pressure on staff nurses. It makes things difficult” (Interviewee 7)*.

“*It is overwhelming for co-workers…a lot of information and communication. For more mature staff nurses who are not used to this new culture, it can be very intimidating” (Interviewee 5)*.

“*A disadvantage of staff nurse empowerment is the expectation that along with our daily work, we do the required scheduling of tasks and goals …” (Interviewee 1)*.

In addition, the nurses reported that communication around change initiatives and projects was not always clear and lacked feedback.

“*A lack of communication between each other and within units, …we were not always informed about what was happening and what had been changed …” (Interviewee 3)*.

*Communication and feedback from nursing councils is limited and they miss the target in my view…” (Interviewee 1)*.

### The nurse manager's role

The respondents reported that the majority of staff nurses were positive about empowerment and increased joint decision-making, but all respondents had also experienced reluctance from some staff nurses. A few study participants noticed that younger staff nurses were more positive about empowerment than were older staff nurses.

“*There were differences. Younger colleagues were more accustomed to empowerment and joint decision-making, other staff nurses were more experienced with empowerment and do well. A few colleagues were difficult to engage—they just wanted to do their jobs…leave them in peace, they weren't interested in participation and saw empowerment as additional demands” (Interviewee 7)*.

Nurse managers noticed that staff nurses' day-to-day workloads were a barrier to achieving increased empowerment. Because of the lack of time in staff nurses' daily practice, direct patient care inevitably took priority over change initiatives and projects.

“*Empowerment needs resources such as enough staff nurses so we can provide time. Work demands and time pressures caused us to fall behind schedule in certain projects. The will is there but because of time pressures we cannot manage it” (Interviewee 8)*.

“*Time investment is necessary and because of time pressures, the engagement needed to take initiative and become involved in decision-making processes gets left behind” (Interviewee 3)*.

“*It is not easy to engage all colleagues given time pressures and high patient care demands” (Interviewee 5)*.

“*I cannot motivate everybody, …I can't manage it sometimes” (Interviewee 6)*.

The respondents reported that physicians were also playing a part in the process, but did not always work inter-professionally because of residual medical departmental structures.

“*Physicians were involved, of course at another level and indirectly …they weren't a barrier. There is still a medical departmental structure that influences our daily practice.” (Interviewee 8)*.

The productive ward program supported physician engagement with empowerment, but not all medical staff were involved. It appeared to be chief physicians, primarily, who were involved with their dedicated nursing units. One nurse manager wished for more support from medical staff.

“*Because of the productive ward rounds module, physicians were consulted and involved. But there is still some distance between us and physicians and they are less engaged” (Interviewee 3)*.

“*Younger physicians less, but our chief physician was engaged and knows what we are doing” (Interviewee 1)*.

“*Too bad in my view, little support and knowledge of what we are doing and why” (Interviewee 4)*.

Hospital management had invested in the empowerment policy by organizing educational and training programs and by providing certain tools.

“*They implemented changes, provided some things, we took training programs and learned to use some tools to get along” (Interviewee 1)*.

Despite the new culture of empowerment, hospital management was still using top-down decision-making.

“*Sometimes hospital management wanted to do certain things…they asked for our opinions but these were not considered” (Interviewee 2)*.

“*Things were moving fast here, and rather thoroughly, and sometimes going a bit too far” (Interviewee 4)*.

Nurse managers reported experiencing discrepancies between the Chief Nursing Officer (CNO) and other executives because of projects the CNO was not aware of. Monthly meetings between the CNO and nurse managers helped to clarify the situation. These meetings were accessible and supported involvement and engagement.

“*We had to do some things and our CNO, our boss, didn't know anything about it. Then I think, how on earth is it possible …” (Interviewee 1)*.

“*Every month we have a meeting with a limited number of nurse managers and our CNO. That makes us feel involved” (Interviewee 8)*.

“*The administration in our hospital is easily accessible. I can express my feelings in the monthly meeting with our CNO” (Interviewee 5)*.

Nurse managers reported that empowerment was not a new idea for them. The majority of the study participants mentioned having introduced empowerment principles previously as part of a unit leadership strategy (e.g., joint decision-making in unit meetings). Three participants referred to social life experiences. Other participants reported learning about empowerment through previous educational and training programs.

“*It was always present in my leadership strategy, …the power comes from our team” (Interviewee 7)*.

“*During unit meetings I always create opportunities for staff nurses to express their opinions, but that's a lower level of empowerment” (Interviewee 2)*.

“*I've had some practice already because I learned about empowerment and involvement through social life experiences” (Interviewee 1)*.

“*I learned about empowerment during an internally organized educational and training program and I still implement those ideas” (Interviewee 4)*.

Two study participants learned about empowerment during their Master's degree program, but explained that putting it into practice was not straightforward.

“*I learned about the concept of empowerment theoretically but practicing those principles wasn't easy. But I knew the concept” (Interviewee 3)*.

Hospital policy surrounding empowerment appears to have had an impact on the role of the nursing unit manager. Three study participants noticed an increase in the amount of work created by the change initiatives and projects they had to supervise.

“*It has an impact on our daily work. We have to do more than in the past—it is not just our patient rounds and staff nurse scheduling. We have to coach our staff nurses about new projects and evaluate those projects too. We have to do a lot by ourselves and that takes time, to discuss things, to evaluate, …it takes time” (Interviewee 6)*.

“*We have to do it, a lot of things were involved, in addition to our patient care, …not only be present at a meeting and report on it, but we have to handle things, search for relevant information, evaluate certain things…we need time for that” (Interviewee 6)*.

“*I have more work to do. It is easier to command and control …probably less effective. But now we have to discuss staff nurses' involvement during meetings, we need more feedback on things, I have to evaluate ongoing projects, I have to support those projects myself too” (Interviewee 1)*.

Two study participants experienced no change in their roles as a result of the new policies supporting empowerment. However, they did have additional tasks to do and also had to learn new skills and tools because of the ongoing projects.

“*The transformation was acceptable for me. I had to learn new skills and learn about the implementation of new tools in the productive ward program. I did not experience huge changes, …it is more intense, of course” (Interviewee 8)*.

One study participant had implemented empowerment principles in the past and experienced no new changes in the nurse manager's role.

“*No huge changes for me. It depends on your leadership strategy,…I always used joint decision-making with my staff nurses about concerns and issues in our daily practice” (Interviewee 4)*.

One participant had only recently been appointed to the nursing unit manager role and was therefore unable to compare the new situation with the past. In total, half of the study participants had to change their nursing unit manager roles because of the policy on supporting empowerment.

“*Often, it is a leadership style, …more responsibility for your team. No more command and control. But you still have to lead a bit, make staff nurses enthusiastic about it. You have to provide support, to engage and maintain staff nurse engagement” (Interviewee 3)*.

“*I try to lead, but it isn't easy, they decide …sometimes it is not the best choice, …but I have to accept that …because my solution was not their choice and they didn't support it …but the second choice was better when supported by joint decision-making. It isn't easy, but I feel it was a better process because we had more respect among our peers” (Interviewee 1)*.

“*It used to be easy, I had it under control, now it is more difficult, I don't know …You have to involve and motivate your team, you have to pull …to meet deadlines. It has changed, just like that, …what I did before was okay, then, not anymore, you have to admit and understand that, okay it is not only my view that counts, …you have to persuade, I have to cope with certain things now, staff nurses' decisions …just as they had to cope with our decisions in the past” (Interviewee 7)*.

One study participant indicated that hospital management had admitted that the nursing unit manager role might be less relevant in the new, flatter type of structure. Other study participants, however, felt that this was not realistic and that the role of the nurse manager is to assure the unit's continuity of care.

“*They (the hospital managers) forgot that nurse managers have an operational role in daily practice, we have to deal with that. For example patient rounds, handing over meetings, training, and coaching new colleagues, …which are tasks we do on a daily basis. Our role is important in assuring the continuity of our unit. They forgot that's our role, you can't ignore that role” (Interviewee 1)*.

### Suggestions by nurse managers to improve the empowerment policy

The participants have suggested a number of initiatives to improve internal hospital governance and the policy surrounding empowerment. For example, they had suggested improvements to communication strategies used in various change initiatives and projects and between healthcare workers. They had also suggested improving the feedback provided to nurse managers by nursing councils.

“*Miscommunication in our hospital was still an issue and sometimes steered by some departments such as IT or technical services. Sometimes there was limited communication between individual healthcare workers and between members of different nursing units too. We were not always aware or informed of certain decisions and changes” (Interviewee 3)*.

“*Limited feedback …such as nursing councils …I didn't know anything about it, …what they were doing or had decided” (Interviewee 1)*.

Secondly, the participants had requested that sufficient support be provided during change initiatives and projects, especially the productive ward program.

“*They promised sufficient support when we started the productive ward program, but it was not always there …you could get it, but you had to work hard to get it” (Interviewee 4)*.

“*When they started the productive ward program there was sufficient support, but later on we had to do it by ourselves. I gave feedback to the management level about the need for support. Sometimes you had to work hard on certain projects and hopefully we were on the right track. Guidelines were clear, but you needed some support, some confirmation” (Interviewee 5)*.

Thirdly, the timing and scheduling of certain projects and the steering by certain departments frustrated some study participants.

“*Our staff nurse scheduling is organized 3 months in advance. Projects that interfered with our scheduling were very frustrating. You can avoid that when projects are planned and scheduled well. If not, it means a lot of unnecessary work” (Interviewee 1)*.

*Project information and initiation comes from different departments with a lot of requests for things that we had to do. It is sometimes overwhelming and …who kept an overall picture of what we have to do?” (Interviewee 4)*.

Two study participants mentioned experiencing time pressure during their daily practice.

“*Staff nurses needed more time to be involved and to participate in certain projects. They were motivated and enthusiastic about it but it is not always possible because of our priority to deliver patient care. When there are expectations you have to create support and resources. We had to ensure that people did not get overloaded” (Interviewee 4)*.

Finally, participants reported that staff nurse involvement was not always in place at the beginning of change initiatives and projects and needed to be evaluated at baseline. The requirements of certain projects ought to be identified and measured before nurses can begin using appropriate tools.

“*There was limited insight and knowledge when projects started—such as the productive ward program—related to whether units were sufficiently ready to start. We need some insight and tools to evaluate readiness before we start certain change initiatives and projects. How can we support readiness to begin certain initiatives? I did it myself a bit and I am very pleased that I did that, to create enough involvement before new projects started” (Interviewee 2)*.

## Discussion

The nurse managers who participated in this study shared their insights into the structural empowerment of staff nurses through organizational components such as internal hospital governance structures and policy, educational and training courses, and certain change initiatives and projects. Staff nurses were involved in joint decision-making processes that fostered engagement, responsibility, autonomy, critical reflection, and communication. The nurse managers reported perceiving these projects and processes as having a positive impact on the quality of care and patient safety. They indicated that the majority of staff nurses were engaged and felt involved in projects such as the productive ward program. These results are in line with a cross-sectional survey conducted among 510 nurses in the Netherlands, which demonstrated the impact of structural and psychological empowerment on innovative behavior; informal power and the extent of impact were found to be the most relevant determinants in the latter study (Knol and van Linge, 2009). Similarly, another survey-based study of 253 mental health staff members found that structural conditions such as opportunity and resources were important for creating support for evidence-based practice (Engström et al., 2015). Our study participants indicated that younger staff nurses were more familiar with the empowerment concept than their older colleagues because of their education and training. Lethbridge et al. (2011) conducted an integrative literature review and described links between structural empowerment, psychological empowerment and reflective thinking as means of assisting undergraduate nursing students to become effective professionals in both their academic and future practice careers.

In this study, nursing unit managers reported that staff nurse empowerment had a certain impact on their managerial roles and, as a result, their experiences proved to be somewhat mixed. Wong and Laschinger (2013) confirmed the mediating role played by nurse empowerment through authentic leadership in nurse performance and job satisfaction. Authentic leadership has been described as “a pattern of transparent and ethical leader behavior that encourages openness in sharing information needed to make decisions while accepting input from those who follow” (Avolio et al., 2009).

Negative aspects of empowerment were also reported in this study; these related mainly to how projects were coordinated and supported and to how the nursing team was prepared for new initiatives in the study hospital. In addition, both staff nurses and nurse managers were experiencing pressure as a result of direct patient care priorities, tightly scheduled projects and miscommunication. A previous Belgian study conducted among 527 nurses showed that long- and short-term sickness absence could be predicted by levels of nursing demand, control and support combined with effort-reward imbalances and over-commitment (Trybou et al., 2014). The authors suggested that nursing administrators should not only monitor and balance nurses' workload and efforts, but also recognize the importance of social support, job control, job rewards, and over-commitment in order to reduce job stress. Our study participants did agree that the CNO played an important supportive role, mainly as a coach and supervisor of change initiatives and projects.

Relationships with physicians in the study hospital were found to be open and supportive, but physicians—with the exception of chief physicians—were felt to be insufficiently aware of the changes being made to foster empowerment. The transition to a flatter, more interdisciplinary organizational structure was still ongoing; this meant that top-down decisions were still being made and a certain degree of departmental interference remained. Study participants had proposed a number of ideas to improve this situation, such as better coordination and communication surrounding change initiatives and projects; provision of dedicated support; monitoring of work demands and priorities; and provision of additional knowledge and tools to ensure nursing unit readiness for new initiatives.

These study results correspond to our previous findings on nurse manager work-related stress and well-being. In the ever-changing, demanding environment of acute hospital nursing units, *role conflict* (e.g., difficulties with competing values, tasks and expectations) and *role meaningfulness* (e.g., feeling involved and significant) have a key impact on emotional exhaustion, work engagement and job satisfaction among nurse managers, as do certain job and organizational characteristics such as time pressure, decision authority, and work agreements (Van Bogaert et al., 2014c).

Study participants reported having mixed feelings about their role and leadership style. The majority agreed that a transformational, participative leadership style should be in place instead of a command and control leadership style. They also reported having doubts about the idea that their roles would be less relevant in the new organizational structure. They felt that the nurse manager role would remain relevant and essential to ensuring the continuity of the unit's operation, both through coordination of projects and through coaching and engaging staff nurses in an empowered unit environment. Clearly, nurse managers have an important leadership role: through staff empowerment, they can simultaneously ensure nurses' health and well-being and the quality of patient care as harmonious objectives.

In line with previous studies, our results show that in order to achieve excellent quality of care and patient safety, it is essential to align short-term and long-term goals among all stakeholders (e.g., physicians, nurse managers, staff nurses, and patients). The health and engagement of front-line workers (e.g., staff nurses and physicians) are vulnerable to negative outcomes such as fatigue, absenteeism, and feelings of cynicism and inefficacy (Khamisa et al., 2013), which may in turn lead to decreased productivity and negative patient outcomes. Empowerment may offer healthcare workers the capacity to decide how care will be organized in order to meet multiple goals. An interdisciplinary, patient-centered approach that includes supportive nurse manager leadership, sufficient training and resources is essential to achieving this. These key conditions will stimulate staff nurse empowerment and support quality improvement as a continuous process, but only when the dynamics and resources of the clinical teams are respected (White, 2015).

### Limitations

The results of this study should be interpreted with caution, as it was performed in one hospital. The manner in which the participants were selected (i.e., nurse managers from medical and surgical units) also means that we have no information about nurse managers' perceptions and experiences of staff nurse empowerment on specialized patient care units such as intensive care and the emergency department. Relatively, new change initiatives were still being implemented in the study hospital at the time of our research; due to time restraints, we were unable to gain a full understanding of their impact on nurse managers and staff nurses and the care they provide.

## Conclusion

Nursing unit managers reported that structural empowerment had had a positive impact on staff nurses' professional attitude and the quality of care and patient safety on their units. Changes in their managerial roles were creating dilemmas in daily practice regarding leadership style. Clear organizational goals and dedicated support for both nurses and nursing unit managers are essential to maintaining an empowered practice environment and thus ensuring optimal care and healthy staff.

## Ethical considerations

All study participants received an invitation letter containing information about the study and an informed consent document. Participant confidentiality was guaranteed. The study was approved by the study hospital's ethics committees.

### Conflict of interest statement

The authors declare that the research was conducted in the absence of any commercial or financial relationships that could be construed as a potential conflict of interest.
